# On the So-called Equine Scarlatina

**Published:** 1883-12-20

**Authors:** Frank S. Billings

**Affiliations:** Vet. Surg., Boston


### ON THE SO-CALLED EQUINE SCARLATINA.

By FRANK S. BILLINGS, VET. SURG., BOSTON.

I have been asked to write a paper on this disease. *There is no such disease.* It has been, and is still, the misfortune of veterinary medicine to be a sort of tail attached to human medicine, a parasite which depends on the latter for strength and support. In the early days of veterinary medicine the nomenclature of the human branch was transferred without reflection to the diseases of animals, as the micro-pathological anatomy of the organs of man has been, and is to-day.

Then we have the "measles" of the hog. Who would for a moment think this was not measles at all, but a cysticercus invasion? We speak of "hog cholera," which bears no relation to the human disease; of the "typhus" of various animals, when nothing resembling the typhus of man, in its peculiar microscopical phenomena, has ever been seen. "Typhoidal" phenomena are common enough in all species of animals, but not those peculiar to typhus.

Veterinary pathologists (we never had one—though many books have been written, we never have yet had a logical, skeptical thinker, as a writer on pathology in veterinary medicine) have yet to learn what constitutes the essentials that should give an animal disease the same name which it occupies in human nosology. To give an animal disease the same name attached to a human disease which it resembles, it must have:

1. The same cause, and be equally transmissible between man and this animal species.
2. Having the same cause, its genus must be similar.
3. The principal points of its pathological anatomy must be similar.
4. It must be similar in course and termination.

These may not be all the points of resemblance, but they are the cardinal ones. and are only slightly modified by the varieties of constitution, anatomical structure of parts, etc. For instance, pneumonia is pneumonia wherever you find it, and so of every organic disease, and in general we have pretty nearly the same varieties in animals as appear in man; but it is singular that the different varieties which occur, under varying circumstances, in man never appear in the same course in our animals, viz.: Broncho-pneumonia is the rule in the horse, cheesy pneumonia in cattle, and catarrhal pneumonia in the dog.

As to "*scarlatina*," to be such in fact, the first necessity would be that it was transmissible in some way from man to the horse, and *vice versa.* This is not the case. It has not one characteristic in common with the disease in man, except that there is a cutaneous eruption. The name has been entirely dropped from *all* the modern continental works on zoopathology, and *never* appears in any of the medical periodicals except those of English origin.

What is known as "scarlatina" is either a condition following on the "influenza diseases," or a new malarial complication due to some new contagium, which the anticipating disease had prepared the way for. It is nothing but a modified form of what is known as "purpura" in English veterinary works, and occurs under exactly the same conditions.

My exact meaning will become clearer to medical readers if I quote from Williams, the best English author. He says:*

"A febrile disease, characterized by an eruption of the skin, petechial spots on the nose, soreness of the throat, and *sometimes* suppuration in different parts of the body, particularly the submaxillary space."

"Unlike the scarlatina which attacks man, it is a non contagious disease, generally attacking but one or two horses in a large stud, among which some form of epizootic disease is at the time prevalent."

"Scarlatina is usually associated with epizootic catarrh, and occurs in animals that have been for some days suffering from that disease; a d the production of such an alteration in the blood as induces the scarlatina is due to defective ventilation or stable drainage, or to overcrowding, by which the air becomes loaded with decomposing animal matter. Sometimes a weak constitution will convert a catarrh into scarlatina, and the severity of an epizootic disease may alter the blood and give origin to scarlatina"

This is from an author who has been called a "world-renowned pathologist." If it is pathology, if there is a grain of logical sense in it, except that the *condition* the author calls *a disease* comes as a sequel of something else, then I do not know the use of language.

The A B C of zoopathology has yet to be written.—*New York Medical Journal.*

